# Bad Effects Sometimes Arising from Taxis in Hernia

**Published:** 1849-06

**Authors:** 


*Bad Effects sometimes arising from Taxis in Hernia.*—In speaking of the taxis, Mr. Gay says, "It is difficult, and even impossible, to obtain any data upon which to calculate the proportion in the total number of deaths after strangulation, which is due to the operation of the manual attempts made to relieve it; or what share these efforts have in the production of the post-mortem appearances in persons in whom the stricture has been relieved by the knife. From the general condemnation of the taxis, almost to proscription, there is every reason to apprehend that much of the fatality in hernia is to be ascribed to it."

Arnaud recites a case in which the taxis was succeeded by suppuration of the omentum. Petit says, "Combien de fois a-t-on vu périr des malades le même jour que la réduction leur a été faite? à l'ouverture des cadavres, on a trouvé aux uns le boyau gangrèneux, aux autres il étoit crevé, et les matières fecales repandues dans le ventre." Other instances are cited by Morand, Bell, Sir. A. Cooper, South, Teale, and others; and it would appear from these that the pathological indications of injury from taxis are peritonitis, gangrene, and rupture of the intestine. "It has fallen to my lot," Mr. Key states, "to see more than one case in which the patient has fallen a victim to a long and continued succession of violent attempts with the taxis. These are followed by a discharge of blood per anum from the bruised vessels of the lining membrane of the gut, which frequently exhausts the patient after the hernia has been returned."

Mr. King has given a tabular view, from unpublished records, of thirty-eight fatal cases of hernia, to illustrate the causes of death, &c.; from which I make the following extract as it relates to the effects of taxis:—

In one the hernia was reduced by taxis; death by peritonitis; gut red.

In one the hernia was reduced by taxis; recovering.

In one the hernia was reduced by taxis, sac and all; the stricture remaining.

In six the hernia was reduced by taxis, but ruptured; death by peritonitis.

Mr. King deduces that nearly one-fourth of the deaths by hernia follow the simple taxis. In one of these cases a reduction "en bloc" was effected by the taxis; and in another, it "was the same thing but for the operation." One patient died from rupture of the bowel, whilst in the other cases death seems to have resulted from peritonitis. The rude employment of the taxis *during an operation*, is, I believe, not unfrequently the cause of death. I have seen an instance of this kind; and am led to think that when the return of the bowel is not promoted by the division of the presumed seat of stricture, and the obstacles are not clear to the operator's mind, the return of the bowel by violence is often preferred to an abandonment of the operation. But in order to rescue the taxis from the opprobrium which its abuse has cast upon it, and to illustrate the advantages which accrue from its judicious employment, I have taken the liberty to construct the following table from Mr. Poland's report of the cases which were treated in Guy's Hospital during the years 1841–42:—We learn from this table, which is too large for insertion, that of 19 cases of hernia, 12 scrotal, 6 femoral, and 10 ventral, only one, a scrotal case, after strangulation had lasted twelve hours, terminated fatally. This table is an abundant plea for its employment, where there are no symptoms of inflammation or other circumstances to interdict it. The question, however, occurs in reference to the taxis, as on a former occasion in reference to delay—why, after the oft-repeated cautions against the contrary mode of its employment, is its use not more constantly limited to the most gentle, and always more succesful, kind of pressure?—*Ibid from Gay on Femoral Hernia.*

